# Multidrug Resistance and Adaptive Response to Silver and Gold Nanoparticles in Methicillin-Resistant *Staphylococcus aureus* from Human and Animal Sources

**DOI:** 10.3390/pathogens15030277

**Published:** 2026-03-04

**Authors:** Eman Marzouk, Mai Ibrahem, Nuha Anajirih, Sulaiman Anagreyyah, Khalid Alamri, Saleh Alamri, Bader Al Hassoun, Abdelmaged Draz, Safiyah Alzahrani, Ayman Elbehiry

**Affiliations:** 1 Department of Public Health, College of Applied Medical Sciences, Qassim University, P.O. Box 6666, Buraydah 51452, Saudi Arabia; e.marzouk@qu.edu.sa; 2 Department of Public Health, College of Applied Medical Science, King Khalid University, Abha 61421, Saudi Arabia; 3 Medical Emergency Services Department, Faculty of Health Sciences, Umm Al-Qura University, Al-Qunfudah 21912, Makkah, Saudi Arabia; 4 Family Medicine Department, King Fahad Armed Hospital, Jeddah 23311, Saudi Arabia; 5 Pharmaceutical Department, Prince Sultan Military Medical City, Riyadh 12233, Saudi Arabia; 6 Department of Veterinary Preventive Medicine, College of Veterinary Medicine, Qassim University, Buraydah 51452, Saudi Arabia

**Keywords:** methicillin-resistant *Staphylococcus aureus*, multidrug resistance, nanoparticles, antimicrobial susceptibility, adaptive resistance, serial sub-inhibitory, public health

## Abstract

Antimicrobial resistance (AMR) remains a serious public health concern, and methicillin-resistant *Staphylococcus aureus* (MRSA) continues to limit treatment options. This laboratory-based comparative study evaluated antibiotic resistance patterns and nanoparticle (NP) susceptibility among 110 *S. aureus* isolates recovered from human skin and soft tissue infections (*n* = 80) and camel milk (*n* = 30). Proteomic identification utilizing matrix-assisted laser desorption ionization time-of-flight mass spectrometry (MALDI-TOF MS) was carried out for all isolates under study. Phenotypic differentiation between MRSA and methicillin-sensitive *S. aureus* (MSSA) was performed via the cefoxitin disk diffusion method, and antimicrobial susceptibility testing was carried out using the disk diffusion method as stated in international guidelines. Multidrug resistance (MDR) was defined by established criteria. The antibacterial activity of silver nanoparticles (AgNPs) and gold nanoparticles (AuNPs) was detected by broth microdilution to determine minimum inhibitory concentration values (MIC_50_ and MIC_90_). The ability to develop reduced susceptibility was evaluated through ten serial sub-inhibitory passages followed by stability testing without using nanoparticles. MRSA prevalence was 52.5% among human isolates and 70% among camel milk isolates. Overall, 56.4% of isolates met MDR criteria, with a significantly higher MDR rate among MRSA compared with MSSA. Both human and camel isolates showed similar resistance patterns. AgNPs exhibited strong antibacterial activity, with MIC_50_ and MIC_90_ values of 0.0078 mg/mL and 0.0156 mg/mL, respectively; nevertheless, AuNPs demonstrated higher MIC values. Response to NPs was similar between isolates, independent of methicillin resistance or MDR. Serial sub-inhibitory exposure resulted in increased MIC values in all tested isolates, and stable resistance persisted in 50% of cases. These results indicate ongoing MRSA circulation in human and animal settings and reinforce the need for careful and controlled use of NP-based antimicrobials.

## 1. Introduction

Antimicrobial resistance (AMR) is widely recognized as a critical global health crisis that substantially undermines the effectiveness of modern medicine [1,2,3]. Methicillin-resistant *Staphylococcus aureus* (MRSA) remains one of the most clinically significant resistant pathogens, causing severe healthcare-associated infections and increasingly common community-associated disease [4,5,6]. Compared with methicillin-susceptible *S. aureus* (MSSA), MRSA infections are associated with greater morbidity, prolonged hospitalization, and increased healthcare costs. These consequences make MRSA surveillance and control a continuing public health priority [7,8].

*S. aureus* colonizes humans and a broad range of animal hosts and can move across ecological boundaries [9,10]. Food-producing animals and animal-derived products may therefore serve as reservoirs of resistant strains and mobile resistance determinants [11,12]. In the Arabian Peninsula, dairy products, particularly camel milk, have been reported to harbor *S. aureus* and MRSA. This creates a potential route for zoonotic exposure and dissemination through the food chain [13,14]. National data from Saudi Arabia confirm persistent MRSA prevalence in clinical settings, with marked regional variation [15,16]. Differences in antimicrobial use, infection control practices, and healthcare infrastructure likely contribute to this variability.

The limited development of new antibiotics has intensified interest in alternative antibacterial strategies. Metallic nanoparticles have attracted attention because of their broad-spectrum activity [17,18,19]. Silver nanoparticles (AgNPs) demonstrate consistent in vitro efficacy against Gram-positive organisms, including MRSA. Their effects involve membrane disruption, oxidative stress, protein damage, and interference with nucleic acids [17,20,21,22]. Gold nanoparticles (AuNPs) exhibit weaker intrinsic antibacterial activity but may exert antimicrobial effects when modified or used as delivery platforms [23,24].

Despite encouraging laboratory findings, emerging evidence indicates that bacterial populations can adapt to NP exposure, particularly under prolonged sub-inhibitory conditions [25,26]. Proposed mechanisms include altered membrane properties, activation of efflux systems, enhanced biofilm formation, and modulation of metal homeostasis pathways [17,26]. These findings challenge the assumption that NPs are inherently resistant-proof and underscore the need to evaluate both immediate efficacy and adaptive risk.

Comparative studies using standardized NP preparations and well-characterized isolates from interconnected human and animal sources remain limited [20]. Robust evaluation requires determination of minimum inhibitory and bactericidal concentrations (MICs and MBCs), assessment of multidrug resistance (MDR) profiles, and controlled serial exposure experiments to detect phenotypic shifts [17,26,27].

The present study was designed to address this gap. We aimed to compare AMR patterns of *S. aureus* isolates recovered from human skin and soft tissue infections (SSTIs) and camel milk within a One Health framework. We further evaluated the in vitro antibacterial activity of AgNPs and AuNPs against these isolates and investigated whether repeated sub-inhibitory exposure could induce stable adaptive changes. By integrating phenotypic resistance profiling with NP susceptibility and adaptation analysis, this study seeks to clarify whether NP activity is influenced by methicillin resistance status, MDR phenotype, or source of origin. These findings are intended to inform the responsible use of NP-based antimicrobials in both clinical and agricultural contexts.

## 2. Materials and Methods

### 2.1. Study Design and Bacterial Isolates

This study was designed as a comparative in vitro investigation to evaluate AMR patterns and NP susceptibility among *S. aureus* isolates from human and animal sources. A total of 110 archived *S. aureus* isolates were included in this study. The term “archived” refers to isolates that were previously collected during routine diagnostic laboratory activities and preserved in CryoBank vials (Mast Diagnostica GmbH, Reinfeld, Germany) at −80 °C after species confirmation. The isolates were not pre-selected based on MRSA or MSSA status. Methicillin resistance classification and antimicrobial susceptibility testing (AST) were performed as part of the present study. Eighty isolates were recovered from human SSTIs, and thirty were obtained from camel milk samples. All isolates originated from routine diagnostic submissions. None were linked to an outbreak or targeted surveillance program.

Human isolates were recovered from 2570 SSTI samples processed at the diagnostic microbiology laboratory of King Faisal Specialist Hospital and Research Centre, Riyadh, Saudi Arabia, between October 2021 and March 2023. From these samples, 87 non-duplicate isolates were initially identified as *S. aureus* using direct microscopy, the API identification system (API^®^ Staph system, bioMérieux SA, Marcy-l’Étoile, France), and the BD Phoenix automated system (BD Phoenix™ M50, Becton, Dickinson and Company, Franklin Lakes, NJ, USA). All 87 isolates were transported to the Microbiology Laboratory, College of Public Health and Health Informatics, Qassim University, Saudi Arabia for confirmatory testing. Species confirmation was carried out using real-time PCR targeting the *nuc* and *mecA* genes and proteomic identification using the Microflex LT MALDI-TOF MS system (Microflex^®^ LT, Bruker Daltonics GmbH, Bremen, Germany). After molecular and proteomic confirmation, 80 isolates were verified as *S. aureus*. Seven isolates were excluded because they did not meet confirmation criteria.

Camel milk isolates were derived from 221 milk samples collected between November 2022 and May 2023 from private veterinary laboratories in the Qassim region, Saudi Arabia. From these samples, 45 isolates were initially identified as *S. aureus* by culture on selective media, coagulase testing, and the MicroScan identification system (MicroScan WalkAway^®^ System, Beckman Coulter Inc., Brea, CA, USA). These isolates were transported to the same university laboratory for molecular and proteomic confirmation using real-time PCR targeting the *nuc* and *mecA* genes and MALDI-TOF MS analysis. Thirty isolates were confirmed as *S. aureus* and included in the study. The remaining isolates were excluded due to failure of confirmation or loss of viability.

All confirmed isolates were preserved in CryoBank™ vials (Mast Diagnostica GmbH, Reinfeld, Germany) and stored at −80 °C. Before experimentation, each isolate was subcultured and re-identified using the Microflex LT MALDI-TOF MS system to ensure species stability after storage. For experimental procedures, isolates were revived on blood agar plates (Oxoid Ltd., Basingstoke, UK) and incubated aerobically at 37 °C for 18 to 24 h. No new human or animal samples were collected for this study. All isolates were anonymized prior to analysis, and no patient-identifiable data were accessed. Ethical approval was not required. All procedures followed institutional biosafety regulations.

### 2.2. Phenotypic Identification of S. aureus

Presumptive identification of *S. aureus* was performed using standard microbiological approaches. Revived isolates were assessed for colony morphology on blood agar plates (Oxoid Ltd., Basingstoke, UK), followed by Gram staining, catalase testing, and coagulase testing. Isolates displaying phenotypic characteristics consistent with *S. aureus* were selected for species-level confirmation.

### 2.3. Species Confirmation by MALDI-TOF Mass Spectrometry

Species identification was confirmed using matrix-assisted laser desorption/ionization time-of-flight mass spectrometry (MALDI-TOF MS), a method widely validated for rapid and accurate bacterial species-level identification [28]. Analysis was performed using a Microflex LT system (Bruker Daltonics GmbH, Bremen, Germany), which has demonstrated high reliability for routine identification of clinical *S. aureus* isolates [29]. Sample preparation followed the direct smear method according to the manufacturer’s instructions. Two to three fresh colonies were applied to a stainless-steel target plate, overlaid with 1 µL of matrix solution (α-cyano-4-hydroxycinnamic acid (HCCA) matrix solution, Bruker Daltonics GmbH, Bremen, Germany), and air-dried prior to analysis. Mass spectra were acquired and compared with the manufacturer’s reference database using Compass Explorer software version 4.1.30. Identification scores of ≥2.0 were considered reliable for species-level identification, in accordance with established validation criteria for MALDI-TOF MS systems. Only isolates confirmed as *S. aureus* were included in subsequent analyses.

### 2.4. Phenotypic Determination of Methicillin Resistance

Methicillin resistance was determined phenotypically using the cefoxitin disk diffusion method as described previously by Skov et al. [30]. Bacterial suspensions were prepared from overnight cultures and adjusted to a 0.5 McFarland standard using sterile saline. The standardized inoculum was spread evenly onto Mueller-Hinton agar plates (Oxoid Ltd., Basingstoke, UK). Cefoxitin disks (30 µg; Oxoid Ltd., Basingstoke, UK) were applied, and plates were incubated aerobically at 35–37 °C for 18–24 h. Inhibition zones were interpreted using CLSI M100 Performance Standards, 33rd edition [31]. Isolates were classified as MRSA or MSSA based on cefoxitin resistance.

### 2.5. Molecular Confirmation by Real-Time PCR

Phenotypic MRSA isolates were confirmed by real-time PCR targeting the *nuc* and *mecA* genes using an Applied Biosystems 7500 Fast Real-Time PCR system (Applied Biosystems™, Thermo Fisher Scientific Inc., Foster City, CA, USA) operated with 7500 Software version 2.3 (Applied Biosystems™, Thermo Fisher Scientific Inc., Foster City, CA, USA). The *nuc* gene was amplified to confirm *S. aureus* species identity, whereas the *mecA* gene was used to verify the genetic basis of methicillin resistance. A genus-specific 16S rRNA primer pair was included as an internal amplification control to confirm the presence of staphylococcal DNA in each reaction.

The primer sequences were as follows: *nuc* forward 5′-TCAGCAAATGCATCACAAACAG-3′ and reverse 5′-CGTAAATGCACTTGCTTCAGG-3′; *mecA* forward 5′-GGGATCATAGCGTCATTATTC-3′ and reverse 5′-AACGATTGTGACACGATAGCC-3′; and 16S rRNA forward 5′-GTGCCAGCAGCCGCGGTAA-3′ and reverse 5′-AGACCCGGGAACGTATTCAC-3′. The expected amplicon sizes were 255 bp for *nuc*, 527 bp for *mecA*, and 886 bp for 16S rRNA.

The *nuc* and *mecA* primer sequences were adopted from the validated protocol described by Dendi et al. [32]. The 16S rRNA primer pair was selected from the protocol reported by Morar et al. [33] for staphylococcal identification. All primers were synthesized commercially (Biomers, Ulm, Germany).

### 2.6. Antibiotic Susceptibility Testing

Antibiotic susceptibility testing was performed using the disk diffusion method on Mueller-Hinton agar (Oxoid Ltd., Basingstoke, UK). Bacterial suspensions were adjusted to a 0.5 McFarland standard and evenly inoculated onto agar plates. The antibiotic panel included cefoxitin (30 µg), penicillin (10 units), erythromycin (15 µg), clindamycin (2 µg), ciprofloxacin (5 µg), gentamicin (10 µg), tetracycline (30 µg), and trimethoprim-sulfamethoxazole (1.25/23.75 µg). All disks were obtained from Oxoid Ltd. (Basingstoke, UK). Plates were incubated at 35–37 °C for 18–24 h. Inhibition zones were measured in millimeters and interpreted according to the CLSI M100 Performance Standards for Antimicrobial Susceptibility Testing, 33rd edition [31]. MDR was defined as resistance to at least one agent in three or more antibiotic classes [34].

### 2.7. Selection and Preparation of NPs

One representative AgNP preparation and one representative AuNP preparation were selected to ensure experimental consistency. Aqueous colloidal AgNPs (10 nm, 0.1 mg/mL) and AuNPs (10 nm, 0.05 mg/mL) were purchased from PlasmaChem GmbH, Berlin, Germany. According to the manufacturer, both NP types were citrate-stabilized spherical particles supplied as stable aqueous suspensions. The 10 nm particle size was selected because smaller NPs provide greater surface area and have demonstrated stronger antibacterial activity in previous in vitro studies, allowing standardized comparison of intrinsic antimicrobial effects [35,36]. Independent physicochemical characterization under experimental conditions, including zeta potential measurement, transmission electron microscopy (TEM) imaging, or hydrodynamic size analysis in Mueller-Hinton broth, was not performed. Nanoparticle properties were based on manufacturer specifications.

### 2.8. Determination of Antibacterial Activity of NPs

The antibacterial activity of AgNPs and AuNPs was evaluated by determining MIC and MBC values using the broth microdilution method, following established AST principles [37]. Serial two-fold dilutions were prepared in sterile 96-well microtiter plates using Mueller-Hinton broth (Oxoid Ltd., Basingstoke, UK). For AgNPs, final concentrations ranged from 0.001 to 0.1 mg/mL. For AuNPs, final concentrations ranged from 0.001 to 0.05 mg/mL. These ranges were selected to encompass sub-inhibitory, inhibitory, and bactericidal concentrations.

Bacterial inocula were prepared from overnight cultures and adjusted to approximately 5 × 10^5^ CFU/mL. Plates were incubated aerobically at 35–37 °C for 18–24 h. The MIC was defined as the lowest NP concentration that completely inhibited visible bacterial growth. For MBC determination, aliquots from wells showing no visible growth were subcultured onto NP-free Mueller-Hinton agar plates and incubated under the same conditions. The lowest concentration yielding no bacterial growth was recorded as the MBC. *S. aureus* ATCC 25923 (American Type Culture Collection, Manassas, VA, USA) was included throughout the study as a quality-control strain to verify the reliability and reproducibility of susceptibility testing procedures. All assays were performed in duplicate and included appropriate growth controls without NPs and sterility controls without bacterial inoculation.

### 2.9. Long-Term Sub-Inhibitory Exposure and Adaptive Response Assessment

Selected MRSA and MSSA isolates from both sources were exposed to sub-inhibitory concentrations of AgNPs or AuNPs to assess potential adaptive responses, following experimental approaches previously described for evaluating NP-induced tolerance and resistance [26]. Twenty isolates were selected to represent MRSA and MSSA from both human and camel sources, including isolates with different resistance profiles, to evaluate adaptive responses across diverse phenotypic backgrounds.

Cultures were incubated for 18–24 h and subsequently passaged into fresh medium containing the same NP concentration. This procedure was repeated for ten consecutive passages to simulate prolonged sub-inhibitory exposure. Susceptibility was reassessed at defined intervals by MIC determination. To evaluate the stability of observed changes, selected isolates were subcultured in NP-free medium prior to repeat testing. Increases in MIC values that persisted after removal of NP selective pressure were interpreted as indicative of stable resistance.

### 2.10. Statistical Analysis

Statistical analyses were performed using appropriate parametric or non-parametric tests based on data distribution. Differences in categorical variables were assessed using the Chi-square test or Fisher’s exact test, as appropriate. Comparisons of MIC values between two independent groups were performed using the Mann–Whitney U test. Correlations between the number of antibiotic classes to which an isolate was resistant and NP MIC values were evaluated using Spearman’s rank correlation coefficient. A *p*-value < 0.05 was considered statistically significant.

## 3. Results

### 3.1. Phenotypic Identification, Species Confirmation, and Source Distribution of S. aureus Isolates

A total of 110 presumptive *S. aureus* isolates were included in the study, comprising 30 isolates from camel milk samples and 80 isolates from human SSTIs. All isolates exhibited phenotypic characteristics consistent with *S. aureus*, including typical colony morphology, Gram-positive staining, and positive catalase and coagulase reactions.

Initial MALDI-TOF MS analysis identified most isolates at the species level, while five isolates yielded identification scores within the 1.700–1.999 range. These included one isolate from camel milk and four isolates from human SSTIs. Following repeat analysis, all five isolates produced species-level identification scores between 2.000 and 2.299. As a result, all isolates were confirmed as *S. aureus*, yielding a 100% species-level confirmation rate.

### 3.2. Prevalence of Methicillin Resistance Among S. aureus Isolates

Phenotypic testing using the cefoxitin disk diffusion method classified all isolates as either MRSA or MSSA. Among the 80 isolates recovered from human SSTIs, 42 isolates (52.5%) were identified as MRSA, while 38 isolates (47.5%) were classified as MSSA. Of the 30 isolates obtained from camel milk samples, 21 isolates (70.0%) were classified as MRSA, and 9 isolates (30.0%) were identified as MSSA.

### 3.3. Antibiotic Susceptibility Profiles and MDR Patterns

Overall resistance rates across the tested antimicrobial classes are summarized in Table 1. Resistance to penicillin was detected in 101 of 110 isolates (91.8%). High resistance rates were also observed for erythromycin (61.8%) and ciprofloxacin (60.9%). Resistance to tetracycline was identified in 49.1% of isolates, followed by clindamycin (33.6%) and gentamicin (28.2%). The lowest resistance rate was recorded for trimethoprim–sulfamethoxazole (17.3%).

When stratified by methicillin resistance status, MRSA isolates exhibited significantly higher resistance rates than MSSA isolates for erythromycin (93.7% vs. 19.1%, *p* < 0.001), clindamycin (46.0% vs. 17.0%, *p* = 0.0029), ciprofloxacin (73.0% vs. 44.7%, *p* = 0.0049), and penicillin (98.4% vs. 83.0%, *p* = 0.0102) (Table 1). No statistically significant differences were observed between MRSA and MSSA for gentamicin (30.2% vs. 25.5%, *p* = 0.7494), tetracycline (50.8% vs. 46.8%, *p* = 0.8252), or trimethoprim–sulfamethoxazole (15.9% vs. 19.1%, *p* = 0.8456).

Comparison by source demonstrated comparable resistance patterns between camel milk and human isolates (Table 1). Penicillin resistance was 86.7% among camel isolates and 93.8% among human isolates (*p* = 0.4142). No significant differences were observed for erythromycin (66.7% vs. 60.0%, *p* = 0.6740), clindamycin (40.0% vs. 31.2%, *p* = 0.5231), ciprofloxacin (63.3% vs. 60.0%, *p* = 0.9206), gentamicin (30.0% vs. 27.5%, *p* = 0.9827), tetracycline (36.7% vs. 53.8%, *p* = 0.1670), or trimethoprim–sulfamethoxazole (16.7% vs. 17.5%, *p* = 1.0000).

### 3.4. MDR Patterns

MDR was defined as resistance to at least one agent in three or more antimicrobial classes, according to the international consensus criteria proposed by Magiorakos et al. [34]. The prevalence of MDR is presented in Table 2. Overall, 62 of 110 isolates (56.4%) met the criteria for MDR. MDR was significantly more frequent among MRSA than MSSA isolates. Forty-eight of 63 MRSA isolates (76.2%) were classified as MDR, compared with 14 of 47 MSSA isolates (29.8%) (*p* < 0.001).

When stratified by source, MDR was identified in 19 of 30 camel milk isolates (63.3%) and 43 of 80 human isolates (53.8%). No statistically significant difference was observed between camel and human isolates (*p* = 0.37).

### 3.5. Antibacterial Activity of AgNPs and AuNPs

The antibacterial activity of AgNPs and AuNPs against *S. aureus* isolates is summarized in Table 3. For AgNPs (10 nm), MIC values ranged from 0.0039 to 0.0313 mg/mL. The overall MIC_50_ and MIC_90_ were 0.0078 mg/mL and 0.0156 mg/mL, respectively. The corresponding MBC range was 0.0078–0.0625 mg/mL. No statistically significant difference was observed between MRSA and MSSA isolates (*p* = 0.412) or between camel milk and human isolates (*p* = 0.538).

For AuNPs (10 nm), MIC values ranged from 0.0156 to 0.125 mg/mL. The overall MIC_50_ and MIC_90_ were 0.0313 mg/mL and 0.0625 mg/mL, respectively. The MBC range was 0.0313–0.250 mg/mL. Differences between MRSA and MSSA isolates were not statistically significant (*p* = 0.621). Similarly, no significant difference was detected between camel milk and human isolates (*p* = 0.447).

**Figure 1:**
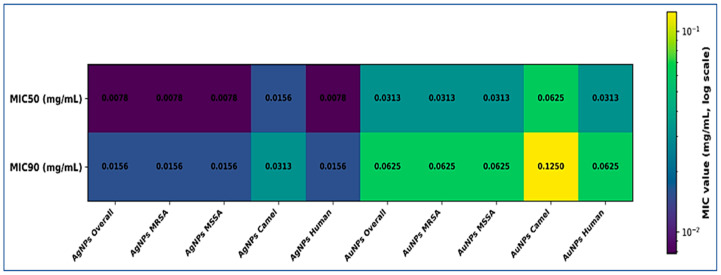
Logarithmic heatmap of MIC_50_ and MIC_90_ values (mg/mL) for AgNPs, 10 nm and AuNPs, 10 nm against *S. aureus* isolates. Subgroups include overall isolates (*n* = 110), MRSA (*n* = 63), MSSA (*n* = 47), camel milk isolates (*n* = 30), and human SSTIs (*n* = 80). Color intensity corresponds to MIC magnitude on a logarithmic scale, with lighter colors indicating lower MIC values and darker colors indicating higher MIC values. Numerical values are displayed within each cell.

Among all isolates, AgNPs demonstrated lower MIC_50_ and MIC_90_ values than AuNPs. However, susceptibility patterns remained comparable between methicillin-resistant and methicillin-susceptible strains and between animal and human sources. The distribution of MIC_50_ and MIC_90_ values among subgroups is visually presented in Figure 1. The logarithmic heatmap illustrates relative differences in NP activity and highlights variation between isolate categories.

### 3.6. Comparison of NP Activity with Antibiotic Resistance Status

Statistical comparisons of NP susceptibility according to antibiotic resistance phenotype are summarized in Table 4. No significant difference in AgNP MIC values was observed between MDR and non-MDR isolates (*p* > 0.05). Similarly, AuNP susceptibility did not differ significantly between MDR and non-MDR groups (*p* > 0.05).

Consistent with findings reported in Section 3.5, MIC values for both AgNPs and AuNPs did not differ significantly between MRSA and MSSA isolates (*p* = 0.412 and *p* = 0.621, respectively). Correlation analysis revealed no significant association between the number of antibiotic classes to which an isolate was resistant and MIC values for either AgNPs or AuNPs (Spearman test, *p* > 0.05).

### 3.7. Adaptive Response Following Serial Sub-Inhibitory NP Exposure

Serial passaging under sub-inhibitory NP concentrations resulted in increased MIC values in all 20 tested isolates (Table 5). Among AgNP-exposed isolates, MIC elevations ranged from two-fold to sixteen-fold. Human MRSA isolates showed increases between four-fold and sixteen-fold, whereas camel-derived MRSA isolates demonstrated smaller shifts ranging from two-fold to four-fold. After removal of AgNP pressure, 3 of 10 isolates (30%) maintained elevated MIC values.

AuNP exposure produced larger MIC shifts. All ten AuNP-exposed isolates exhibited at least an eight-fold increase, and four isolates demonstrated sixteen-fold elevations. Following NP withdrawal, 7 of 10 AuNP-exposed isolates (70%) retained elevated MIC values.

Overall, persistent elevation of MIC values after serial exposure was observed in 10 of 20 isolates (50%), indicating phenotypic stability of reduced susceptibility following removal of NP pressure. The difference between AuNP and AgNP groups (70% vs. 30%) did not reach statistical significance (χ^2^ = 3.2, *p* = 0.073). In isolates in which MIC values declined after NP removal, the changes were interpreted as reversible phenotypic adaptation rather than stable persistence.

## 4. Discussion

AMR continues to compromise the treatment of hospital and community infections. This study evaluated *S. aureus* isolates from clinical and camel milk sources within a One Health framework. Three key findings emerged. First, MRSA prevalence was high in both human and camel isolates. Second, MDR was common and significantly associated with MRSA. Third, AgNPs and AuNPs showed measurable antibacterial activity, but prolonged sub-inhibitory exposure resulted in adaptive shifts.

More than half of the human skin and soft tissue isolates were MRSA at 52.5%. The proportion was even higher in camel milk isolates at 70%. These values are aligned with national surveillance and meta-analyses reporting persistent MRSA prevalence in Saudi Arabia, with regional variation across the country [15,16]. The detection of MRSA in camel milk is particularly important. Previous investigations have identified MRSA in food-producing animals and dairy products, including pasteurized camel milk [14,38,39]. These findings support the view that animal-derived products may serve as reservoirs of resistant strains. They also emphasize the need for integrated surveillance linking human, veterinary, and food systems [14,38].

All isolates were confirmed as *S. aureus* using MALDI-TOF MS. This method provides rapid and reliable species identification and reduces time to diagnosis compared with conventional techniques [40]. Several studies have shown that MALDI-TOF spectral patterns may assist in differentiating MRSA from MSSA using analytical models [41]. Alternative matrix strategies and spectral preprocessing have improved discrimination between resistant and susceptible isolates [42]. Systematic reviews have identified spectral markers associated with methicillin resistance, including PSM-mec and delta toxin peaks [43]. MALDI-TOF has also been validated for identification of foodborne pathogens, supporting its use for dairy-associated isolates [44]. These data support the reliability of species identification in both human and camel isolates included in this study.

MDR was common, with 56.4% of isolates meeting international MDR criteria. MDR was significantly more frequent among MRSA isolates at 76.2% compared with 29.8% in MSSA. This difference reflects the known capacity of MRSA lineages to accumulate additional resistance determinants [45]. Similar findings have been reported in hospital and community settings [46].

Resistance to erythromycin and ciprofloxacin was high, particularly among MRSA isolates. This pattern is in agreement with regional and international data showing increased macrolide and fluoroquinolone resistance in MRSA compared with MSSA [46,47]. Penicillin resistance exceeded 90% among isolates. This finding corresponds to the widespread distribution of beta-lactamase production in *S. aureus* [48]. Importantly, resistance rates did not differ significantly between camel and human isolates. MDR prevalence was also nearly identical between sources. These similarities indicate that resistance determinants circulate among human and animal compartments under shared antimicrobial pressures [49,50]. Globally, increasing AMR has been documented in both clinical and food-animal environments. Regions with variable antimicrobial stewardship and high antibiotic use in human and veterinary medicine are particularly affected [51].

AgNPs showed strong antibacterial activity. The MIC_50_ and MIC_90_ values were 0.0078 mg/mL and 0.0156 mg/mL. These concentrations fall within previously reported in vitro ranges for AgNPs against *S. aureus*, including MDR strains [21,52]. Susceptibility to AgNPs did not differ between MRSA and MSSA isolates. In addition, NP MIC values were not correlated with the number of antibiotic resistance classes. These results suggest that classical antibiotic resistance mechanisms do not confer cross-protection against AgNPs. AgNPs are known to disrupt bacterial membranes, induce oxidative stress, and interfere with essential cellular processes [21]. These mechanisms differ from conventional antibiotic targets.

AuNPs showed higher MIC values than AgNPs, indicating weaker intrinsic antibacterial activity. This observation is consistent with evidence that AuNP efficacy depends largely on surface functionalization and particle characteristics rather than core metallic properties [19,53]. Nevertheless, AuNP susceptibility was also independent of methicillin resistance and MDR phenotype.

The most important finding of this study concerns adaptive shifts during serial sub-inhibitory exposure. After ten passages under NP pressure, all tested isolates showed increased MIC values. Persistent phenotypic reduced susceptibility after NP withdrawal was observed in 50% of isolates. The proportion was higher after AuNP exposure at 70% compared with 30% after AgNP exposure. Although this difference did not reach statistical significance, the trend is relevant. Previous studies have shown that bacteria can develop reduced susceptibility or stable adaptive responses after prolonged exposure to metallic NPs [25,26].

Several mechanisms have been proposed to explain NP adaptation. These include altered membrane permeability, activation of efflux systems, enhanced biofilm formation, and changes in metal homeostasis pathways [25,54]. Biofilm formation may reduce effective NP contact with bacterial cells [55,56]. These mechanisms provide a plausible explanation for the MIC increases observed in this study.

The larger MIC shifts observed after AuNP exposure may reflect differences in interactions between nanoparticles and bacterial cells. AgNPs often exert stronger immediate bactericidal effects due to ion release and membrane damage. In contrast, sub-lethal AuNP exposure may allow sufficient survival for adaptive responses to emerge [57]. The persistence of elevated MIC values after removal of NP pressure suggests phenotypic stability of reduced susceptibility. However, molecular mechanisms were not investigated, and mutation-driven resistance cannot be confirmed. Similar concerns have been raised regarding reduced susceptibility following prolonged NP exposure [58]. These findings suggest that metallic NPs may also be subject to evolutionary pressure under sustained exposure conditions.

From a One Health perspective, the inclusion of both human and camel isolates highlights the ecological continuity of *S. aureus* among clinical and food-animal settings [38,59]. However, the present findings reflect phenotypic similarity only, and without molecular genotyping (e.g., WGS, MLST, spa typing, or SCCmec characterization), clonal linkage between human and camel isolates cannot be established. Comparable resistance patterns and NP susceptibility profiles further support the presence of overlapping antimicrobial pressures within interconnected systems rather than confirmed strain circulation [5,38].

AgNPs remain promising adjunct agents against MDR MRSA due to their multi-target antibacterial activity and reported synergy with conventional antibiotics [60,61]. However, the adaptive shifts observed in this study highlight the need for careful dosing strategies. Prolonged sub-therapeutic exposure in clinical, agricultural, or environmental contexts may promote reduced susceptibility [25,62]. Responsible implementation requires monitoring frameworks, safety assessment, and regulatory guidance to limit unintended selection pressure [18,63]. These findings emphasize that nanoparticle-based antimicrobials should be incorporated within stewardship frameworks similar to conventional antibiotics.

In summary, this study demonstrates high MRSA prevalence in both clinical and camel milk isolates, substantial MDR with strong ecological overlap, potent antibacterial activity of AgNPs, and clear evidence that prolonged sub-inhibitory NP exposure can induce adaptive shifts. The observed similarity between human and camel isolates should be interpreted as phenotypic overlap within a shared ecological framework, not as evidence of confirmed transmission. These findings support cautious and responsible use of NP-based antimicrobials within a One Health framework.

## 5. Limitations

Several limitations should be taken into consideration when interpreting these findings. First, isolates were obtained from defined clinical and food-associated sources within a limited geographic setting. Although the results provide relevant regional insight, they may not represent the broader epidemiology of *S. aureus* in other regions or production systems. In addition, the sample size was limited to the number of eligible and confirmed isolates available during the defined collection period, which may restrict broader generalization of the findings.

Second, the AST panel was limited to commonly used and epidemiologically relevant agents. Last-line anti-MRSA agents such as vancomycin, linezolid, daptomycin, and teicoplanin were not evaluated, and broth microdilution testing was not performed for expanded therapeutic profiling. Therefore, the study does not provide comprehensive clinical susceptibility data for advanced treatment options.

Third, comprehensive molecular genotyping such as multilocus sequence typing, clonal complex determination, SCCmec typing, or spa typing was not performed. Consequently, potential associations between specific genetic lineages and observed resistance patterns could not be evaluated. Therefore, the study cannot determine genetic relatedness or confirm transmission dynamics between human and camel isolates. In addition, the presence of heavy metal resistance genes or SCC-associated metal resistance elements was not investigated.

Fourth, the adaptive exposure experiments were conducted on a representative subset of isolates rather than the full strain collection. Inclusion of a larger number of clinical isolates and additional reference strains would strengthen future investigations of nanoparticle-induced adaptation.

Fifth, NP susceptibility and adaptive exposure experiments were conducted under controlled in vitro conditions. These experimental settings do not replicate complex in vivo environments, where host factors, immune responses, and microbial interactions may influence antimicrobial activity.

An additional limitation is that NP characterization was based on manufacturer specifications. Surface charge, confirmation of morphology by TEM, and stability in Mueller-Hinton broth were not independently evaluated. These properties may influence NP dispersion, interaction with bacterial cells, and antimicrobial activity. Further physicochemical characterization under experimental conditions would allow more accurate interpretation of interactions between NPs and bacterial cells.

Finally, the molecular mechanisms underlying the observed adaptive shifts were not investigated. The persistence of elevated MIC values was defined phenotypically based on repeat susceptibility testing after NP withdrawal and does not confirm genetic mutation or permanent resistance. Genomic or transcriptomic analysis would be required to determine whether the observed increases in MIC values were associated with specific mutations, regulatory changes, or population-level selection dynamics.

## 6. Conclusions

This study confirms a high prevalence of MRSA in both human SSTIs at 52.5% and camel milk isolates at 70%. These findings show that resistant *S. aureus* circulates in both clinical and food-associated settings. MDR was substantial, with 56.4% of isolates meeting international MDR criteria and a significantly higher frequency among MRSA compared with MSSA. Similar resistance patterns in camel and human isolates support the presence of shared selective pressures within a One Health context. However, molecular typing would be required to confirm clonal relatedness or direct transmission between these compartments. AgNPs demonstrated strong in vitro antibacterial activity, with lower MIC_50_ and MIC_90_ values than AuNPs. Susceptibility to NPs was not associated with methicillin resistance or MDR status, indicating that their antibacterial action differs from conventional antibiotic mechanisms. However, repeated sub-inhibitory exposure resulted in increased MIC values in all tested isolates, and persistent phenotypic reduced susceptibility was observed in 50% of cases after NP withdrawal. The higher proportion of persistent phenotypic reduced susceptibility following AuNP exposure raises concern about adaptive selection under prolonged low-level exposure. These findings indicate that metallic NPs may also undergo selective pressure under sustained exposure conditions. Overall, this study highlights persistent MRSA burden between interconnected human and animal reservoirs, confirms the antibacterial potential of AgNPs, and emphasizes the need for careful and controlled application to avoid unintended resistance development.

## Figures and Tables

**Table 1:** Antibiotic resistance rates among *S. aureus* isolates according to source and methicillin resistance status.

Antibiotic	Antibiotic Class	Overall		MRSA		MSSA		*p*-Value (MRSA vs. MSSA)	Camel		Human		*p*-Value (Camel vs. Human)
		*n*	%	*n*	%	*n*	%		*n*	%	*n*	%	
Penicillin	β-lactam (penicillin)	101	91.8%	62	98.4%	39	83.0%	0.0102	26	86.7%	75	93.8%	0.4142
Erythromycin	Macrolide	68	61.8%	59	93.7%	9	19.1%	0.0000	20	66.7%	48	60.0%	0.6740
Clindamycin	Lincosamide	37	33.6%	29	46.0%	8	17.0%	0.0029	12	40.0%	25	31.2%	0.5231
Ciprofloxacin	Fluoroquinolone	67	60.9%	46	73.0%	21	44.7%	0.0049	19	63.3%	48	60.0%	0.9206
Gentamicin	Aminoglycoside	31	28.2%	19	30.2%	12	25.5%	0.7494	9	30.0%	22	27.5%	0.9827
Tetracycline	Tetracycline	54	49.1%	32	50.8%	22	46.8%	0.8252	11	36.7%	43	53.8%	0.1670
Trimethoprim–sulfamethoxazole	Folate pathway inhibitor	19	17.3%	10	15.9%	9	19.1%	0.8456	5	16.7%	14	17.5%	1.0000

**Table 2:** Prevalence of MDR among *S. aureus* isolates.

Group	MDR		Non-MDR		Total (*n*)	*p*-Value
	*n*	%	*n*	%		
Overall (*n* = 110)	62	56.4%	48	43.6%	110	-
MRSA (*n* = 63)	48	76.2%	15	23.8%	63	<0.001 *
MSSA (*n* = 47)	14	29.8%	33	70.2%	47	-
Camel milk (*n* = 30)	19	63.3%	11	36.7%	30	0.37
Human isolates (*n* = 80)	43	53.8%	37	46.2%	80	-

*p*-values represent comparisons between MRSA and MSSA, and between camel and human isolates where applicable. * Statistically significant at *p* < 0.05.

**Table 3:** Antibacterial activity of AgNPs and AuNPs against *S. aureus* isolates.

NP	Group	MIC Range (mg/mL)	MIC_50_ (mg/mL)	MIC_90_ (mg/mL)	MBC Range (mg/mL)	*p*-Value (MRSA vs. MSSA)	*p*-Value (Camel vs. Human)
AgNPs (10 nm)	Overall (*n* = 110)	0.0039–0.0313	0.0078	0.0156	0.0078–0.0625	0.412	0.538
	MRSA (*n* = 63)	0.0039–0.0313	0.0078	0.0156	0.0078–0.0625		
	MSSA (*n* = 47)	0.0039–0.0313	0.0078	0.0156	0.0078–0.0625		
	Camel (*n* = 30)	0.0078–0.0313	0.0156	0.0313	0.0156–0.0625		
	Human (*n* = 80)	0.0039–0.0313	0.0078	0.0156	0.0078–0.0625		
AuNPs (10 nm)	Overall (*n* = 110)	0.0156–0.125	0.0313	0.0625	0.0313–0.250	0.621	0.447
	MRSA (*n* = 63)	0.0156–0.125	0.0313	0.0625	0.0313–0.250		
	MSSA (*n* = 47)	0.0156–0.125	0.0313	0.0625	0.0313–0.250		
	Camel (*n* = 30)	0.0313–0.125	0.0625	0.125	0.0625–0.250		
	Human (*n* = 80)	0.0156–0.125	0.0313	0.0625	0.0313–0.250		

**Table 4:** Statistical comparison of NP susceptibility according to antibiotic resistance phenotype.

NP	Comparison	*n*	*p*-Value
AgNPs (10 nm)	MDR vs. non-MDR	110	>0.05
	MRSA vs. MSSA	110	0.412
AuNPs (10 nm)	MDR vs. non-MDR	110	>0.05
	MRSA vs. MSSA	110	0.621
AgNPs (10 nm)	Correlation (No. resistant classes vs. MIC)	110	>0.05
AuNPs (10 nm)	Correlation (No. resistant classes vs. MIC)	110	>0.05

Footnote: *p*-values for group comparisons were calculated using the Mann–Whitney U test. Correlations were assessed using Spearman’s rank correlation.

**Table 5:** Changes in MIC values following serial sub-inhibitory NP exposure among selected MDR *S. aureus* isolates (*n* = 20).

Isolate ID	Source	Phenotype	NP	Baseline MIC (mg/mL)	MIC After 10 Passages (mg/mL)	Fold Increase	MIC After NP Removal (mg/mL)	Persistent Phenotypic Reduced Susceptibility
H1	Human	MRSA	AgNPs	0.0019	0.0156	8-fold	0.0156	Yes
H2	Human	MRSA	AgNPs	0.0019	0.0313	16-fold	0.0156	No
H3	Human	MRSA	AgNPs	0.0019	0.0156	8-fold	0.0078	No
H4	Human	MRSA	AgNPs	0.0019	0.0078	4-fold	0.0039	No
H5	Human	MRSA	AgNPs	0.0019	0.0156	8-fold	0.0156	Yes
H6	Human	MSSA	AuNPs	0.0078	0.0625	8-fold	0.0625	Yes
H7	Human	MSSA	AuNPs	0.0078	0.0625	8-fold	0.0313	No
H8	Human	MSSA	AuNPs	0.0078	0.0625	8-fold	0.0625	Yes
H9	Human	MSSA	AuNPs	0.0078	0.0625	8-fold	0.0313	No
H10	Human	MSSA	AuNPs	0.0078	0.125	16-fold	0.125	Yes
C1	Camel	MRSA	AgNPs	0.0039	0.0078	2-fold	0.0039	No
C2	Camel	MRSA	AgNPs	0.0039	0.0078	2-fold	0.0039	No
C3	Camel	MRSA	AgNPs	0.0039	0.0156	4-fold	0.0156	Yes
C4	Camel	MRSA	AgNPs	0.0039	0.0156	4-fold	0.0078	No
C5	Camel	MRSA	AgNPs	0.0039	0.0078	2-fold	0.0039	No
C6	Camel	MSSA	AuNPs	0.0078	0.0625	8-fold	0.0625	Yes
C7	Camel	MSSA	AuNPs	0.0078	0.0625	8-fold	0.0625	Yes
C8	Camel	MSSA	AuNPs	0.0078	0.125	16-fold	0.125	Yes
C9	Camel	MSSA	AuNPs	0.0078	0.0625	8-fold	0.0313	No
C10	Camel	MSSA	AuNPs	0.0078	0.125	16-fold	0.125	Yes

Serial passaging was performed for ten successive passages at sub-inhibitory NP concentrations. Fold increase was calculated as MIC after passage divided by baseline MIC. Persistent phenotypic reduced susceptibility was defined as persistence of elevated MIC after removal of NP pressure.

## Data Availability

The raw data supporting the conclusions of this article will be made available by the authors on request.
